# All-Optical XOR, AND, OR, NOT, NOR, NAND, and XNOR Logic Operations Based on M-Shaped Silicon Waveguides at 1.55 μm

**DOI:** 10.3390/mi15030392

**Published:** 2024-03-13

**Authors:** Amer Kotb, Kyriakos E. Zoiros, Wei Chen

**Affiliations:** 1 School of Chips, XJTLU Entrepreneur College (Taicang), Xi’an Jiaotong-Liverpool University, Taicang, Suzhou 215400, China; 2 Department of Physics, Faculty of Science, University of Fayoum, Fayoum 63514, Egypt; 3 Lightwave Communications Research Group, Department of Electrical and Computer Engineering, School of Engineering, Democritus University of Thrace, 67100 Xanthi, Greece; kzoiros@ee.duth.gr

**Keywords:** optical logic operations, silicon-on-silica waveguide, contrast ratio

## Abstract

Silicon waveguides are essential to integrated photonics, which is where optical and electronic components are coupled together on a single silicon chip. These waveguides allow for the integration of signal processing and optical transmission, which advances data centers, telecommunications, and other optical applications. Thus, our study involves the simulation of essential all-optical logic operations, namely XOR, AND, OR, NOT, NOR, NAND, and XNOR, and utilizes M-shaped silicon optical waveguides at a wavelength of 1.55 μm. This simulation is conducted through Lumerical FDTD solutions. The suggested waveguide comprises four identical slots, configured in the shape of the letter ‘M’, and all of which are formed of core silicon and silica cladding. These logic operations work based on constructive and destructive interferences that are caused by phase changes in the input optical beams. The contrast ratio (CR) is employed to quantitatively and comparatively assess the degree to which the target logic operations are efficiently executed. The simulation results indicate that, compared to other reported designs, the considered logic functions constructed using the proposed waveguide can be implemented with higher CRs. The outcomes of this paper can be utilized regarding the implementation of optoelectronic combinational logic circuits of enhanced functionality.

## 1. Introduction

Silicon (Si) is widely utilized in the electronics industry as a semiconductor, making it a crucial material in the production of computer chips, solar cells, waveguides, etc. Silica refers to silicon dioxide (SiO_2_), a compound made up of silicon and oxygen atoms. It is one of the most abundant compounds on Earth and is found in various forms, such as quartz, sand, and agate. Si waveguides, on the other hand, are typically made of silicon-on-insulator (SOI) substrates, where a thin layer of silicon is sandwiched between layers of SiO_2_. These waveguides guide light through the Si core by exploiting the refractive index contrast between Si and the surrounding SiO_2_. The high refractive index of Si ensures that the light is confined within the Si core, preventing it from spreading into the cladding. Si waveguides are applied in various fields, including telecommunications, data communications, optical interconnects, and sensors. They play a crucial role in connecting different components on a photonic integrated circuit, such as lasers, modulators, detectors, and other passive elements. Additionally, Si waveguides offer several advantages, including compatibility with existing CMOS technology, which enables cost-effective manufacturing. They also allow for the integration of photonics with existing electronic integrated circuits on a single chip, creating opportunities for more efficient and compact devices [1,2,3,4,5,6,7,8]. On the other hand, optical logic operations can have potential advantages in terms of speed and energy efficiency as compared to traditional electronic circuits [9,10]. Indeed, optical logic gates have recently been designed and/or constructed utilizing a variety of waveguide designs [11,12,13,14,15,16,17,18,19,20,21,22,23,24,25,26,27,28,29,30,31,32,33,34,35,36,37,38,39,40,41]. Implementing complete optical computing systems based on optical logic functions is a challenging task because it involves maintaining signal integrity over long distances, dealing with issues such as signal loss and dispersion, and developing practical devices that can be integrated into larger systems. The compact Si waveguide that we propose in this work is capable of executing seven optical logic operations (XOR, AND, OR, NOT, NOR, NAND, and XNOR) at 1.55 μm simultaneously, while the majority of the other reported designs have used photonic crystals (PhCs) [11,12,13,14,15,16,17,18,19,20,21,22] or noble metals [32,33,34,35,36,37] to implement only one or, at most, two logic operations. Four identical slots, configured in the shape of the letter ‘M’, comprise the suggested waveguide; these slots are all composed of Si on top of SiO_2_. Compared to our pervious Si designs [26,27,28,29,30,31], this proposed waveguide is easier to use in the communications network, as well as in the design of many computational logic circuits. The variety of waveguide designs allows engineers and designers to choose the most appropriate structure for a given application, taking into account factors like frequency range, mode handling, size, polarization, materials, manufacturing constraints, and specialized requirements. This versatility enables the optimization of performance and efficiency in a wide range of electromagnetic wave-based systems. In this paper, the logic operations work is based on constructive interference (CI) and destructive interference (DI) that results from the input optical beams’ phase variations. The performance of the logic operations is evaluated utilizing the contrast ratio (CR) metric [26,27,28,29,30,31] through Lumerical finite-difference-time-domain (FDTD) solutions [42], with the convolutional perfectly matched layer (PML) as an absorbing boundary condition [43]. The incident light will be absorbed with a minimal number of reflections under the PML absorbing boundary conditions. However, spurious numerical reflections occur in a discretized FDTD space even when the PML is perfectly capable of absorbing incident beams. The coordinate stretching variables inside the PML might be graded to lessen these reflections [43]. In this case, the XYZ axis mesh accuracy is set to 0.05 μm, 0.05 μm, and 0.01 μm, respectively. The simulation findings show that the examined logic functions generated when using the suggested waveguide can be achieved with higher CRs when compared to other reported designs [11,16,17,18,22,23,34,35,40,41] (see Table 8). These findings complement and generalize our relevant research that has been conducted so far [26,27,28,29,30,31], and essentially extends the technological suite in the quest for providing different design and building options for Si waveguide-based core logic modules.

## 2. Waveguide Principle

**Figure 1:**
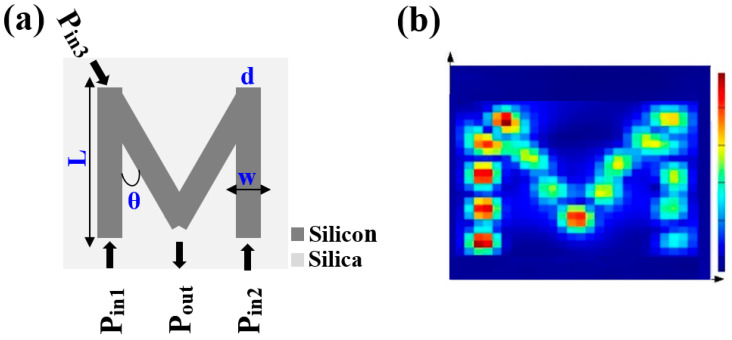
(**a**) Schematic illustration and (**b**) electric field intensity distributions of M-shaped Si waveguide.

Figure 1 depicts the schematic illustration and electric field intensity distributions of the proposed waveguide, which is made up of four identical slots configured in the shape of the letter ‘M’, all of which are implemented on an SOI platform of a core Si layer and SiO_2_ cladding. All slots have a fixed length (L) of 1.0 μm, width (w) of 0.22 μm, thickness (d) of 0.3 μm, and angle (θ) of 60°. The FDTD simulations have been run iteratively until we were strongly confident about their applicability and validity.

The spectral transmission (T) of Si depends on the thickness of the Si wafer and the specific type of Si (e.g., crystalline or amorphous). Crystalline Si has good transmission in the infrared region, but it starts to absorb light in the visible and ultraviolet regions. A transverse magnetic (TM) mode polarized pulse at 1.55 μm excites the input ports. The outcomes of the simulations are captured by setting FDTD monitors, i.e., $T=I_{out}/I_{in}{=\left|E_{out}\right|}^{2}/{\left|E_{in}\right|}^{2}$, where I_out_ is the intensity at the output port (i.e., port 4), and $I_{in}=I_{1}+I_{2}+I_{3}$, which is the sum of the intensities at three input ports [26,27,28,29,30,31]. The value of 0.2 is assigned to the normalized threshold transmission (T_th_), which is the minimum normalized power required to generate T. To optimize T, the incident beams have to meet suitable phase-matching conditions [44,45]. From a logical perspective, port 4 produces an output of ‘1’ only if T > T_th_ and ‘0’ if T ≤ T_th_.

The interaction between silicon waveguides and an incident optical signal is typically described by the phenomenon of mode coupling [46,47,48]. Mode coupling refers to the interaction between different guided modes within the waveguide structure. When an incident signal encounters a waveguide, it can excite various guided modes depending on factors such as wavelength, polarization, and the waveguide’s geometric and refractive index properties. In the context of silicon waveguides, the interaction involves the coupling of the incident signal to specific guided modes supported by the waveguide. These guided modes can include fundamental modes (e.g., TE or TM modes) and higher-order modes, each with its unique spatial distribution of the electric field within the waveguide. Understanding and controlling mode coupling is crucial for designing efficient photonic devices and circuits. Engineers and researchers use numerical simulations, as well as experimental techniques, to analyze and optimize mode coupling in silicon waveguides for specific applications, such as signal routing, modulation, waveguides, and detection in integrated optical systems.

**Figure 2:**
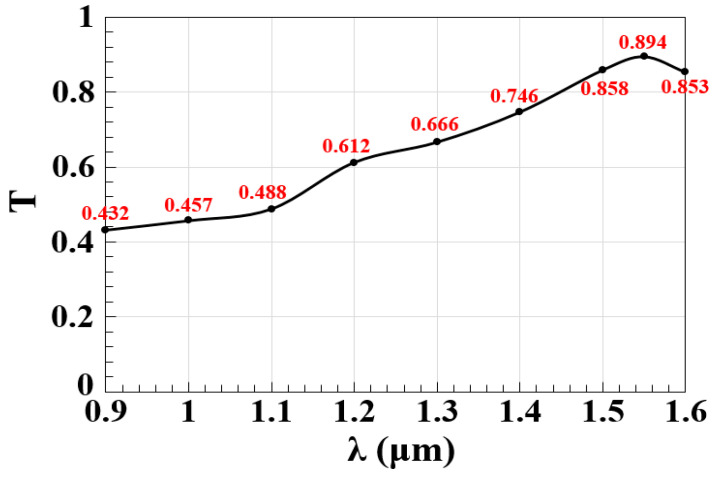
T versus λ using the M-shaped Si waveguide at 1.55 μm.

In Figure 2, the illustration specifically showcases how the normalized spectral transmission (T) of the M-shaped silicon waveguide varies with the operating wavelength (λ), assuming that all incident beams are uniformly launched at the three input ports with the same phase of 180°. At the wavelength of 1.55 μm, the proposed waveguide demonstrates an impressive high T of 0.894. A more detailed examination of the figure emphasizes that this waveguide consistently operates with high T over a broader wavelength spectrum, spanning from 1.2 μm to 1.6 μm. This extended range underscores the versatility and reliability of the waveguide’s performance across different wavelengths, making it a promising candidate for various optical applications.

**Figure 3:**
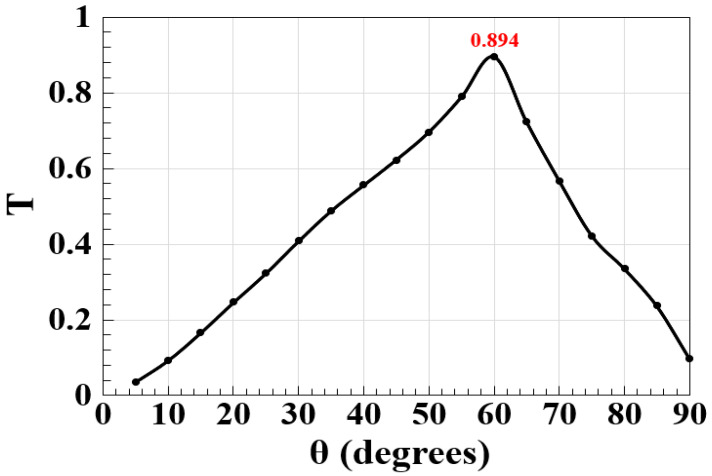
T versus θ using the M-shaped Si waveguide at 1.55 μm.

The angle between slots (i.e., θ) may affect how light or signals are manipulated within the waveguide. The effect of θ on T at 1.55 μm is thus simulated in Figure 3. Upon the examination of this figure, it is evident that the maximum T = 0.894 is achieved at θ = 60°. Consequently, this is the optimum value specified for θ, which is accordingly used as fixed throughout our simulations. Expanding on this figure, it is also evident that altering the value of θ results in an increase in the light scattering and absorption within the materials, thereby reducing T.

**Figure 4:**
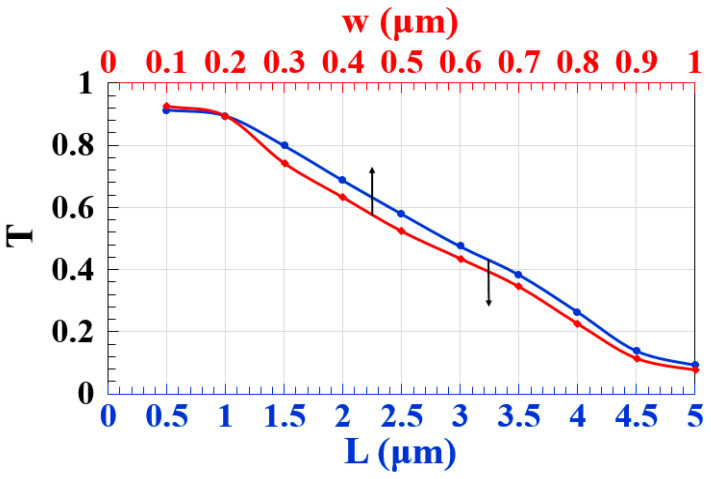
T versus L and w using the M-shaped Si waveguide at 1.55 μm.

The dimensions of a Si waveguide can significantly impact its performance, especially in the context of integrated photonics. For example, the length of the waveguide influences the overall propagation losses. Longer waveguides tend to have higher losses, and minimizing the length is often crucial for device efficiency. A more narrow waveguide tends to have lower losses [49]. Therefore, Figure 4 illustrates how the slot length (L) and width (w) affect T. As seen in this figure, high T is produced by the suggested Si waveguide across a broad range of L and w, or L = 0.5–2.5 µm and w = 0.1–0.5 µm.

## 3. Logic Operations

### 3.1. XOR, AND, OR

**Figure 5:**
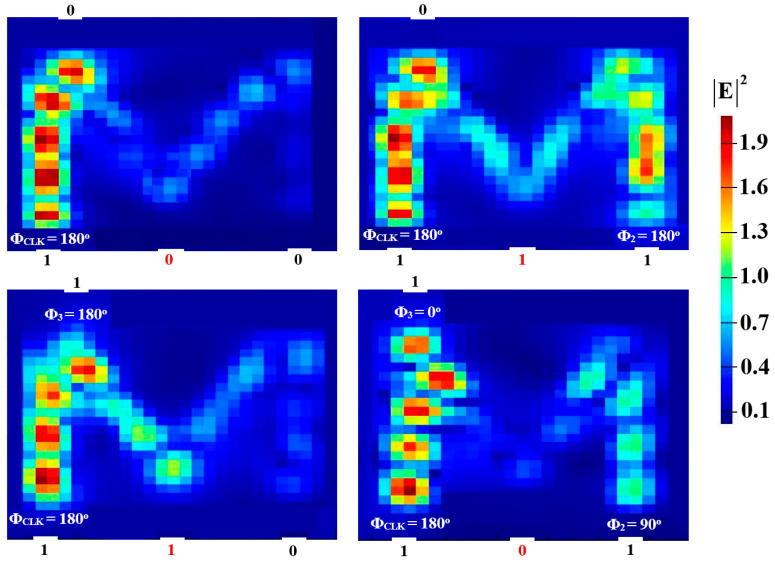
XOR field intensity distributions using the M-shaped Si waveguide at 1.55 μm.

**Figure 6:**
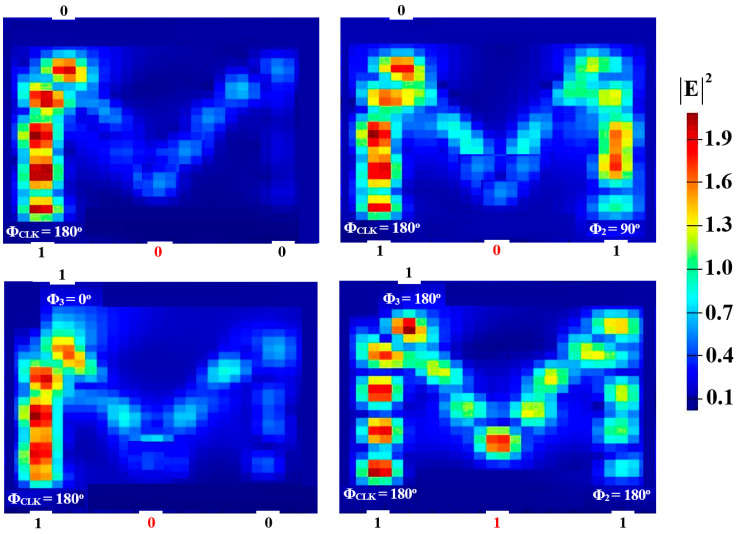
AND field intensity distributions using the M-shaped Si waveguide at 1.55 μm.

**Figure 7:**
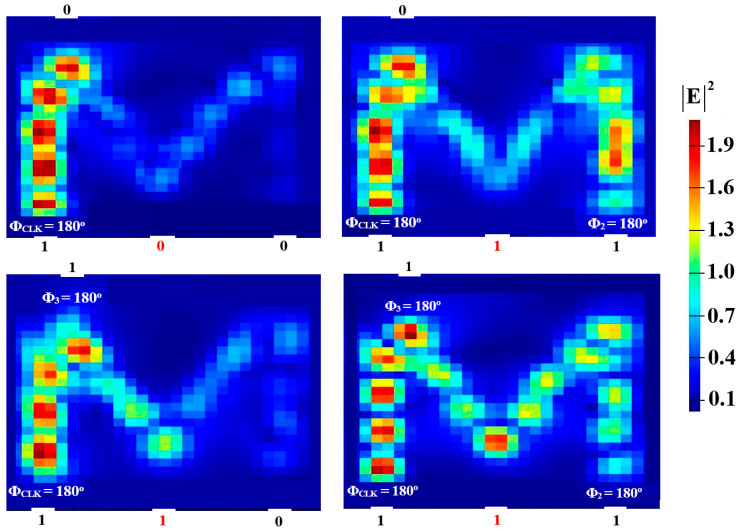
OR field intensity distributions using the M-shaped Si waveguide at 1.55 μm.

Two input beams are injected into P_in2_ and P_in3_, and P_in1_ must supply a clock beam (CLK) with an angle of 180^o^ to execute the XOR, AND, and OR logic operations. Establishing a reference phase difference between input beams that results in either CI or DI can be done using the CLK (all ‘1’s). A ‘1’ output is produced by CI when all input beams are launched with the same phase, but a ‘0’ output is produced by DI when these beams exhibit different phases. It is noteworthy to observe that the output logic operation occurs in between the two signals launched into the proposed waveguide from P_in2_ and P_in3_. Using the M-shaped Si waveguide at 1.55 μm, the field intensity distributions of the XOR, AND, and OR operations are shown in Figure 5, Figure 6 and Figure 7, respectively.

The suggested waveguide proved successful in achieving a high CR. The simulation results for the AND, OR, and XOR logic operations at 1.55 μm in terms of T and CR are summarized in Table 1, Table 2 and Table 3, respectively.

### 3.2. NOT, NOR, NAND, XNOR

**Figure 8:**
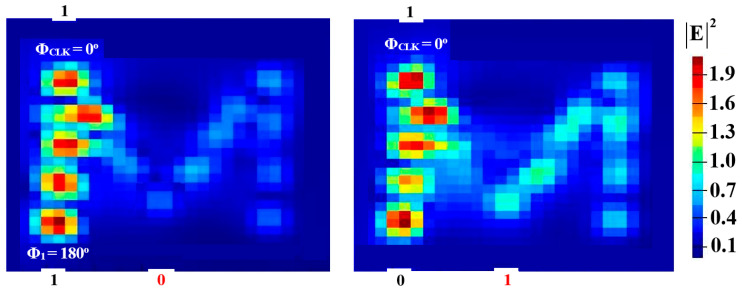
NOT field intensity distributions using the M-shaped Si waveguide at 1.55 μm.

**Figure 9:**
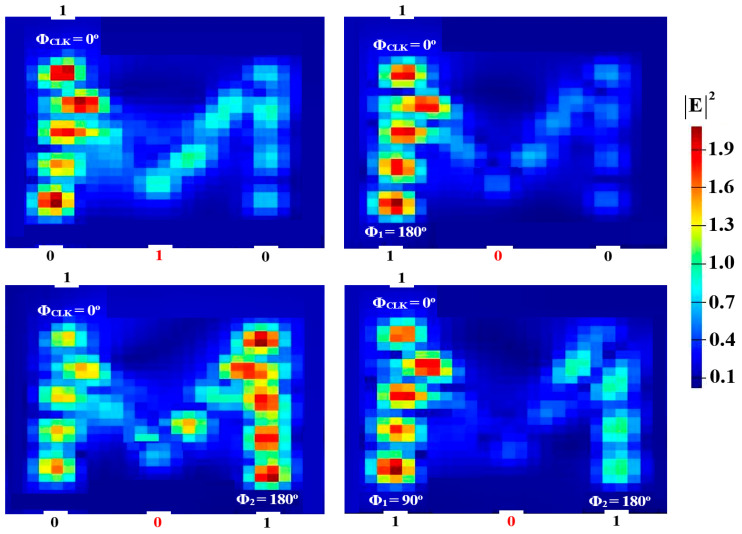
NOR field intensity distributions using the M-shaped Si waveguide at 1.55 μm.

**Figure 10:**
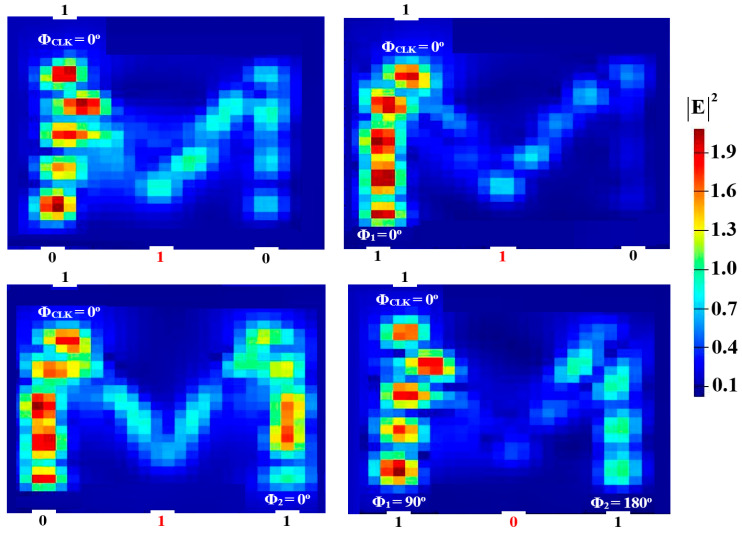
NAND field intensity distributions using the M-shaped Si waveguide at 1.55 μm.

**Figure 11:**
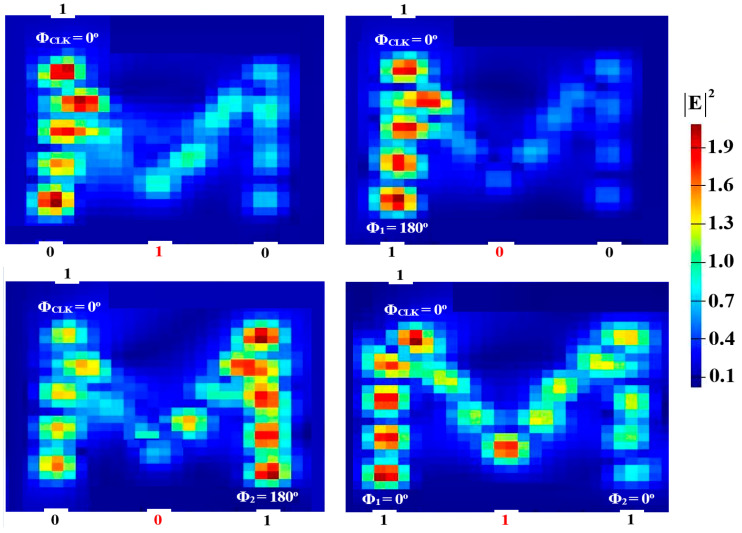
XNOR field intensity distributions using the M-shaped Si waveguide at 1.55 μm.

In order to perform NOT, NOR, NAND, and XNOR logic operations, a Clk with Φ_Clk_ = 0° is injected into P_in3_, while two beams are injected into P_in1_ and P_in2_ (see Figure 1). When the input beams are launched at different angles, they interact destructively and incur a logical ‘0’ at P_in4_. However, when launched at identical angles, they interfere constructively and make a logical ‘1’ appear at P_in4_. Figure 8, Figure 9, Figure 10 and Figure 11 display the field intensity distributions of the NOT, NOR, NAND, and XNOR operations, respectively, using the suggested Si waveguide at 1.55 μm.

Table 4, Table 5, Table 6 and Table 7 provide a summary of the simulation findings for the NOT, NOR, NAND, and XNOR operations at 1.55 μm, respectively. These tables indicate that our design achieves acceptable performance.

A comparison of our waveguide with the other published designs-based optical logic operations [11,16,17,18,22,23,34,35,40,41] is given in Table 8. This comparison includes both theoretical and practical results using various optical structures. This table proves that the suggested design has several advantages, such as being created with cost-effective materials like Si and SiO_2_, as well as being more compact, and providing a better performance. Additionally, with the possibility of designing using electron beam lithography or laser techniques, the suggested design enables seamless integration with electronic components on a single chip. These advantages make our compact waveguides a popular choice in the development of integrated photonic circuits for other optical applications.

Fabricating nanometer-scale waveguides demands sophisticated techniques in microfabrication and nanotechnology. Key methodologies, such as advanced photolithography (e.g., extreme ultraviolet lithography or electron beam lithography), are pivotal for crafting silicon-on-silica waveguides. Achieving high precision and control in optical structure creation is facilitated by femtosecond direct writing. Etching processes, like reactive ion etching or deep reactive ion etching, play a crucial role in precisely shaping silicon and forming the intricate waveguide structures. The deposition of thin silicon layers on silica substrates can be achieved through either chemical vapor deposition or physical vapor deposition. It’s worth noting that the selection of specific techniques depends on the unique requirements of the waveguide design, as well as the available fabrication facilities. In this dynamic field, staying abreast of the latest research and developments in nanotechnology and microfabrication is imperative. It is noteworthy that ongoing advancements in these technologies continually unfold, emphasizing the importance of remaining current with emerging methodologies. Despite the intricacies involved, the experimental validation of the proposed waveguide is within reach due to the accessibility of an advanced manufacturing processes. Rather than presenting an insurmountable obstacle, the fabrication challenge becomes a practical matter with potential solutions. Furthermore, recent reports highlight successful experimental implementations of multiple optical logic gates based on various optical waveguides. These findings, documented in references [23,32,34,50,51,52,53,54,55,56,57,58], signify a significant stride forward and pave the way for analogous implementations in the future. The footprint of other waveguide-based implementations of optical logic gates is even smaller, as reported, for instance, in reference [40] and cited in the corresponding column of Table 8.

## 4. Conclusions

We have proposed and demonstrated, through Lumerical FDTD simulations, all-optical XOR, AND, OR, NOT, NOR, NAND, and XNOR logic operations at 1.55 μm using four identical slots configured in the shape of the letter ‘M’, all of which are formed of core Si and SiO_2_ cladding. The constructive and destructive interferences that result from the phase shifts, induced by the inserted input optical beams, constitute the fundamental principles for the physical realization of these logic operations. According to the theoretical findings, the suggested waveguide allows for the construction of the target logic operations while exhibiting higher CRs than other reported designs. The outcomes of this work can open new possibilities for the practical design and implementation of optoelectronic combinational logic circuits of advanced functionality.

## Figures and Tables

**Table 1:** XOR simulation results (T_th_ = 0.20).

Φ_CLK_	Φ_2_	Φ_3_	P_out_	T	CR (dB)
180°	-	-	0	0.024	14.94
180°	180°	-	1	0.586	
180°	-	180°	1	0.786	
180°	90°	0°	0	0.021	

**Table 2:** AND simulation results (T_th_ = 0.20).

Φ_CLK_	Φ_2_	Φ_3_	P_out_	T	CR (dB)
180°	-	-	0	0.024	15.36
180°	90°	-	0	0.028	
180°	-	0°	0	0.026	
180°	180°	180°	1	0.894	

**Table 3:** OR simulation results (T_th_ = 0.20).

Φ_CLK_	Φ_2_	Φ_3_	P_out_	T	CR (dB)
180°	-	-	0	0.024	15.20
180°	180°	-	1	0.586	
180°	-	180°	1	0.786	
180°	180°	180°	1	0.894	

**Table 4:** NOT simulation results (T_th_ = 0.20).

Φ_1_	Φ_CLK_	P_out_	T	CR (dB)
180°	0°	0	0.024	15.10
-	0°	1	0.775	

**Table 5:** NOR simulation results (T_th_ = 0.20).

Φ_1_	Φ_2_	Φ_CLK_	P_out_	T	CR (dB)
-	-	0°	1	0.775	15.28
180°	-	0°	0	0.024	
-	180°	0°	0	0.026	
90°	180°	0°	0	0.021	

**Table 6:** NAND simulation results (T_th_ = 0.20).

Φ_1_	Φ_2_	Φ_CLK_	P_out_	T	CR (dB)
-	-	0°	1	0.775	14.65
0°	-	0°	1	0.575	
-	0°	0°	1	0.484	
90°	180°	0°	0	0.021	

**Table 7:** XNOR simulation results (T_th_ = 0.20).

Φ_1_	Φ_2_	Φ_CLK_	P_out_	T	CR (dB)
-	-	0°	1	0.775	15.24
180°	-	0°	0	0.024	
-	180°	0°	0	0.027	
0°	0°	0°	1	0.894	

**Table 8:** Comparison of proposed and other waveguide-based waveguides of optical logic functions.

References	Functions	Design	Materials	Size (μm^2^)	Wavelength (nm)	CR (dB)
[11]	AND, XOR, OR, NOT, NAND, NOR XNOR	PhC waveguides	Si/air	9 × 5	1550	5.42–9.59
[16,17,18]	AND, XOR, XNOR	T-shaped PhC waveguides	Si/air	-	1550	8.29–33.05
[22]	AND, OR	2D PhC design	Si/air	19.8 × 12.6	1520	9.74 and 17.95
[23]	AND, NOR, XNOR	Si photonics platform			1550	>10 dB
[34]	NOT, XOR, AND, OR, NOR, NAND, XNOR	Metal slot waveguide	Silver/SiO_2_	1.5 × 2.36	632.8	6–16
[35]	NOT, XOR, AND, OR, NOR, NAND, XNOR	Metal-insulator-metal structures	Air/silver	5.33 × 0.42	632.8	15
[40]	AND, NAND, OR, XOR, NOR, XNAOR, NOT	Plasmonic logic gates design	Silver/SiO_2_	0.25 × 0.25	850	4.14–14.46
[41]	AND, OR, NOT, NAND	Inverse design on Si platforms	Si/SiO_2_	1.0 × 1.5	1300	0.5–5.79
This work	XOR, AND, OR, NOT, NOR, XNOR, NAND	M-shaped Si waveguides	Si/SiO_2_	1.0 × 1.0	1550	14.65–15.36

## Data Availability

Data are contained within the article.
